# Supplementary figures and images for: Correlation of patient symptoms with SARS-CoV-2 Omicron variant viral loads in nasopharyngeal and saliva samples and their influence on the performance of rapid antigen testing
Source: Microbiol Spectr. 2024 Oct 9;12(11):e00932-24. doi: 10.1128/spectrum.00932-24 (PMC11536993; doi:10.1128/spectrum.00932-24)

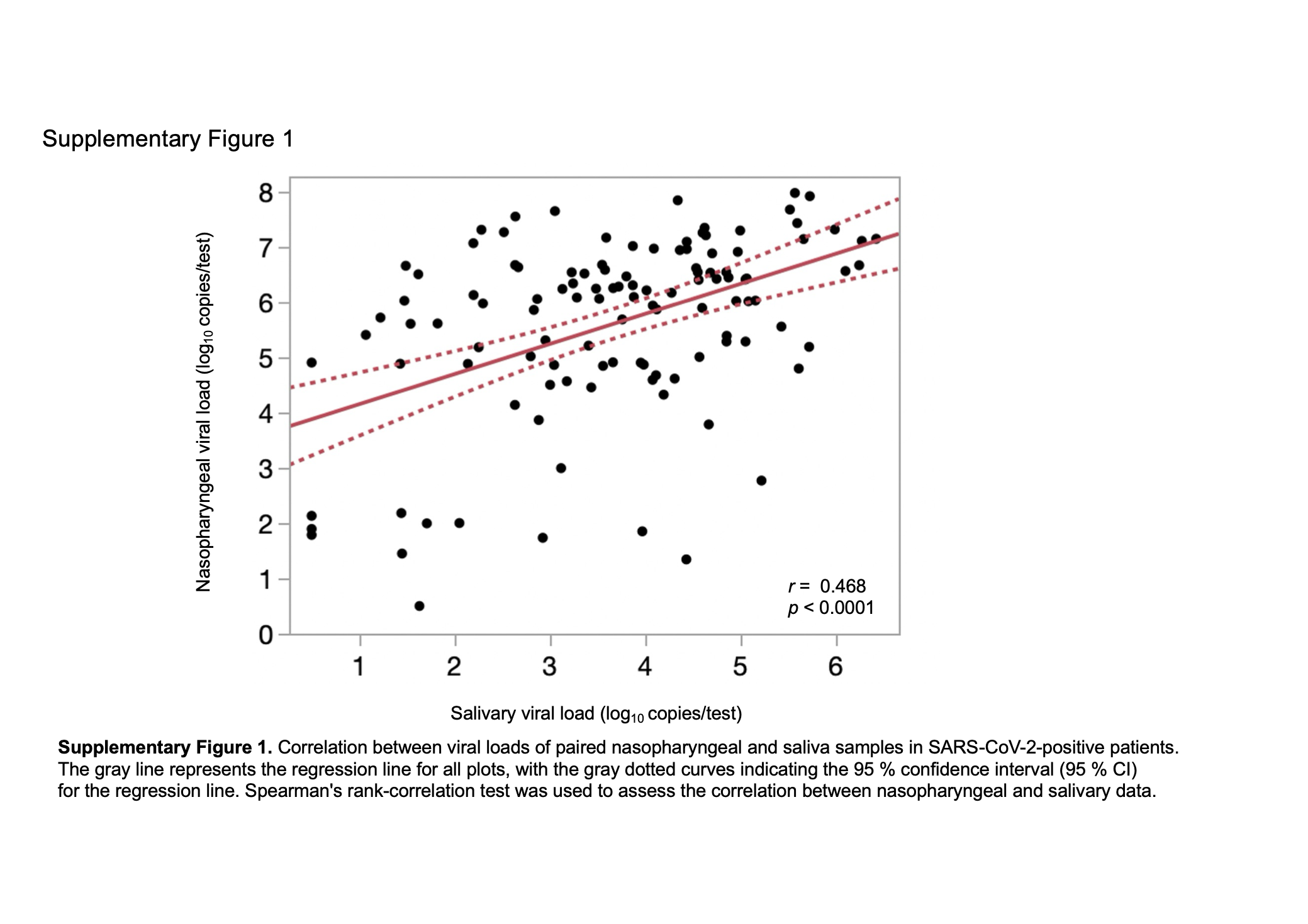

Supplement: Figure S1 — Correlation between viral loads of paired nasopharyngeal and saliva samples in SARS-CoV-2-positive patients. [file spectrum.00932-24-s0001.tiff]
